# A baculoviral system for the production of human β-glucocerebrosidase enables atomic resolution analysis

**DOI:** 10.1107/S205979832000501X

**Published:** 2020-05-29

**Authors:** Rhianna J. Rowland, Liang Wu, Feng Liu, Gideon J. Davies

**Affiliations:** a Department of Chemistry, York Structural Biology Laboratory, University of York, Heslington, York YO10 5DD, United Kingdom; b Department of Chemistry, University of British Columbia, Vancouver, British Columbia V6T 1Z1, Canada

**Keywords:** β-glucocerebrosidase, Gaucher disease, insect–baculovirus expression, crystallization

## Abstract

The lysosomal glycoside hydrolase β-glucocerebrosidase (GBA; sometimes called GBA1 or GC_ase_) catalyses the hydrolysis of glycosphingolipids. Inherited deficiencies in GBA cause the lysosomal storage disorder Gaucher disease (GD). Consequently, GBA is of considerable medical interest, with continuous advances in the development of inhibitors, chaperones and activity-based probes. The development of new GBA inhibitors requires a source of active protein; however, the majority of structural and mechanistic studies of GBA today rely on clinical enzyme-replacement therapy (ERT) formulations, which are incredibly costly and are often difficult to obtain in adequate supply. Here, the production of active crystallizable GBA in insect cells using a baculovirus expression system is reported, providing a nonclinical source of recombinant GBA with comparable activity and biophysical properties to ERT preparations. Furthermore, a novel crystal form of GBA is described which diffracts to give a 0.98 Å resolution unliganded structure. A structure in complex with the inactivator 2,4-dinitrophenyl-2-deoxy-2-fluoro-β-d-glucopyranoside was also obtained, demonstrating the ability of this GBA formulation to be used in ligand-binding studies. In light of its purity, stability and activity, the GBA production protocol described here should circumvent the need for ERT formulations for structural and biochemical studies and serve to support GD research.

## Abstract 2

Active human β-glucocerebrosidase was produced in baculovirus-transduced insect cells, purified and crystallized to yield a 0.98 Å resolution unliganded structure and a trapped covalent intermediate structure with 2-deoxy-2-fluoro-β-d-glucopyranoside.

## 1. Introduction   

**Figure 1:**
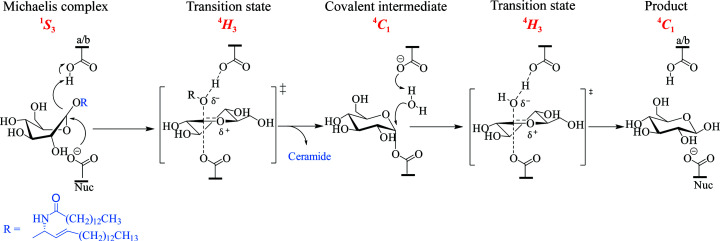
GBA hydrolyses glucosylceramide in a two-step double-displacement mechanism to yield ceramide and glucose with retention of β-stereochemistry.

β-Glucocerebrosidase (glucosylceramidase; GBA; EC 3.2.1.45; often termed GBA1 or GC_ase_) is a membrane-associated lysosomal enzyme belonging to the GH30 family of glycoside hydrolases (http://www.cazy.org; Lombard *et al.*, 2014 ▸). GBA is responsible for catalysing the hydrolysis of glycosphingolipids, specifically glucosylceramide (GlcCer), by the hydrolytic cleavage of β-linked glucose moieties from sphinogolipid aglycones (Brady *et al.*, 1965 ▸). Mechanistically, GBA is a retaining β-glucosidase which hydrolyses its substrates with net retention of β-anomeric stereochemistry (mechanisms are reviewed in Gloster & Davies, 2010 ▸; Zechel & Withers, 2000 ▸). Retention of anomeric configuration is achieved through the Koshland double-displacement mechanism (Koshland, 1953 ▸) using two key carboxylate-containing residues; in the case of human GBA, Glu340 serves as the catalytic nucleophile and Glu235 acts as the general acid/base (Fig. 1 ▸).

Inherited deficiencies in GBA activity cause an accumulation of GlcCer within lysosomes, subsequently leading to the most common lysosomal storage disorder, Gaucher disease (GD; Grabowski & Horowitz, 1997 ▸; Brady *et al.*, 1966 ▸). Traditionally, GD is classified into three clinical phenotypes based on the presence of neurological manifestations and the rate of neuronopathic disease progression (Zhao & Grabowski, 2002 ▸). The non-neuropathic form, GD type 1, is the most common clinical type, accounting for over 90% of GD cases (Mistry *et al.*, 2011 ▸; Mehta, 2006 ▸). Types 2 and 3 are less common neuronopathic forms of GD involving the central nervous system (CNS), with a continuum of phenotypes ranging from death *in utero* to slowly progressive CNS deterior­ation over decades (Davies *et al.*, 2007 ▸; Erikson *et al.*, 1997 ▸). Consequently, GBA is of significant clinical importance.

Enzyme-replacement therapy (ERT) was introduced in the early 1990s as a first-line treatment for GD. The aim of ERT is to correct for the underlying GBA deficiency and alleviate clinical symptoms by supplying patients with active exogenous enzyme (Beck, 2018 ▸; Mistry & Abrahamov, 1997 ▸). In 1991, Ceredase (alglucerase), a glyco-modified GBA formulation purified from human placenta (Barton *et al.*, 1990 ▸, 1991 ▸; Furbish *et al.*, 1977 ▸), was approved for ERT. Subsequently, the Genzyme Corporation (Sanofi Genzyme, Cambridge, Massachusetts, USA) developed Cerezyme (imiglucerase), a recombinant formulation with mannose-terminated N-glycans produced in a Chinese hamster ovary (CHO) cell line (Serratrice *et al.*, 2016 ▸; Deegan & Cox, 2012 ▸). However, Cerezyme production was halted in 2009 by a vesivirus infection at Genzyme’s production facility (Qiu *et al.*, 2013 ▸), promoting the development of alternative ERTs. In 2010, velaglucerase alfa (VPRIV; Shire HGT, Lexington, Massachusetts, USA), a recombinant product produced by specific gene activation in an HT-1080 cell line, was licenced for use (Ben Turkia *et al.*, 2013 ▸; Brumshtein *et al.*, 2010 ▸). Later, taliglucerase alfa (Elelyso; Pfizer, New York, USA), a plant-derived variant expressed in carrot-root cells, was approved in the US (Zimran *et al.*, 2016 ▸; Aviezer *et al.*, 2009 ▸; Shaaltiel *et al.*, 2007 ▸).

Given the hydrophobic nature of GBA and the post-translational glycosylation that is required to ensure proper folding, GBA cannot be produced in prokaryotic systems (Grace & Grabowski, 1990 ▸). Consequently, eukaryotic systems with the necessary post-translational modification capabilities must be employed, as exemplified by the existing ERT expression systems. In addition to these expression platforms, GBA production has been attempted in murine cells (Fabrega *et al.*, 2000 ▸), COS-1 cells (Grabowski *et al.*, 1989 ▸), seeds of the *Arabidopsis thaliana* plant (He *et al.*, 2012 ▸), glycoengineered *Nicotiana benthamiana* plants (Limkul *et al.*, 2016 ▸), *Pichia pastoris* (Sinclair & Choy, 2002 ▸) and baculoviral expression vector systems (BEVS; Martin *et al.*, 1988 ▸; Sinclair *et al.*, 2006 ▸; Sawkar *et al.*, 2006 ▸). Such diversity demonstrates the current lack of consensus on a robust and economical platform for GBA production for nonclinical use. Accordingly, there is a considerable ongoing reliance on expired ERT formulations for biochemical, mechanistic and structural studies. These ERT preparations can be incredibly costly, and are often only obtainable under a Material Transfer Agreement (MTA) and in limited supply. Given the clinical importance of GBA and the continuing development of novel GBA chaperones (Goddard-Borger *et al.*, 2012 ▸; Diot *et al.*, 2011 ▸; Hill *et al.*, 2011 ▸; Marugan *et al.*, 2011 ▸), inhibitors (Artola *et al.*, 2019 ▸; Kuo *et al.*, 2019 ▸; Zoidl *et al.*, 2019 ▸; Schröder *et al.*, 2018 ▸) and activity-based probes (ABPs; Artola *et al.*, 2017 ▸, 2019 ▸; Schröder *et al.*, 2018 ▸; Beenakker *et al.*, 2017 ▸), there is a pressing need for reliable sources of recombinant GBA to meet research demands and reduce the reliance upon ERT formulations.

The use of baculovirus expression vectors was first described in the 1980s and has since proved to be useful for the production of many recombinant proteins (Chambers *et al.*, 2018 ▸; Bonning & Hammock, 1996 ▸; Cameron *et al.*, 1989 ▸; Luckow & Summers, 1988 ▸). The *Autographa californica* multicapsid nucleopolyhedrovirus (AcMNPV) of the *Baculoviridae* family is the most applied baculoviral vector for recombinant protein production (Jarvis, 2003 ▸; Possee, 1997 ▸; Jarvis *et al.*, 1996 ▸; Blissard & Rohrmann, 1990 ▸). Lepidopteran insect-cell lines, such as *Spodoptera fruigiperda* (Sf9 cells) and *Trichoplusia ni* (BTI-Tn-5B1-4, Tn5, High Five cells), have been studied extensively as hosts for this viral vector (Bieniossek *et al.*, 2012 ▸; Fitzgerald *et al.*, 2006 ▸; Wilde *et al.*, 2014 ▸; Jarvis *et al.*, 1990 ▸). However, to our knowledge, no insect-cell-produced GBA has been approved for therapeutic use because the glycoforms produced by insect cells can be immunogenic (Hancock *et al.*, 2008 ▸). Of note, High Five cells modify glycoproteins with a core α-1,3-fucose, which can induce immunogenic and allergic responses in humans. Several strategies have been developed to address the issue of incompatible N-glycosylation in insect-cell expression systems; however, these remain in their infancy (Zitzmann *et al.*, 2017 ▸).

Previous studies on the use of BEVS platforms for GBA production have shown some success, albeit with inconsistent results regarding protein quantity and quality. Early work by Martin *et al.* (1988 ▸) using the AcMNPV vector and polyhedrin promoter in Sf9 cells demonstrated GBA expression, with enzymatic activity detected in both the cell extract and culture medium. However, the efficiency of protein secretion was called into question in subsequent studies (Berg-Fussman *et al.*, 1993 ▸; Grabowski *et al.*, 1989 ▸). Additionally, Choy *et al.* (1996 ▸) demonstrated that GBA produced in AcMNPV-transfected Sf9 cells can be stored intracellularly. More recently, Sinclair *et al.* (2006 ▸) employed the *Orgyia pseudo­tsugata* multicapsid nucleopolyhedrovirus (OpNPV) for GBA production in Sf9 cells to investigate the effect of the full-length and shortened native signal sequences on GBA secretion. The full-length signalling construct was reported to produce 30% more enzymatic activity than the shortened construct but, in contrast to previous studies (Xu & Grabowski, 1998 ▸; Choy *et al.*, 1996 ▸), both constructs resulted in the secretion of the majority of the GBA into the medium (Sinclair *et al.*, 2006 ▸). In addition, wild-type GBA and an N370S GD-associated mutant have been expressed in High Five cells (Sawkar *et al.*, 2006 ▸; Wei *et al.*, 2011 ▸) to evaluate the stability of the protein and its susceptibility to chaperone-mediated stabilization; however, no information on protein yield or secretion efficiency have been reported for this expression system. Despite this success, little has since been published with regard to the production of GBA using BEVS, and there remains a lack of literature describing the establishment of a reliable baculoviral GBA expression platform for academic research purposes.

As part of our long-standing interest in the development of ABPs to study GBA and other glycosidases, we established a BEVS platform for the production of recombinant GBA for biochemical and structural studies. Here, we describe the production of active human GBA in insect cells using a MultiBac AcNPV-derived expression system (Bieniossek *et al.*, 2012 ▸), which circumvents the need for commercial sources of GBA. In this approach, Sf9 cells were used to generate the recombinant baculovirus, whilst High Five cells were employed for the production of recombinant GBA. An N-terminally truncated GBA gene, lacking its native signalling sequence, was used in conjunction with the honeybee melittin signal sequence (Tessier *et al.*, 1991 ▸), a widely used secretion signal in insect-cell expression, to drive the secretion of recombinant GBA into the cell medium. This recombinant GBA was active against the artificial substrate 4-methyl­umbelliferyl-β-d-glucopyranoside and exhibited an optimum thermal stability at pH 5.2, as expected for a lysosomal enzyme. Furthermore, our recombinant GBA was purified with a typical yield of 3.6–4.6 mg per litre of cell medium, providing sufficient protein for biochemical and structural analysis. Consequently, our GBA was studied by X-ray crystallography to generate a 0.98 Å resolution unliganded structure in a novel crystal form. This is the highest resolution structure of GBA deposited to date, allowing exquisite atomic resolution analysis of GBA that reveals two conformations of the catalytic acid/base residue. A structure in complex with the β-glucosidase inactivator 2,4-dinitrophenyl-2-deoxy-2-fluoro-β-d-glucopyranoside was also obtained, demonstrating the utility of this recombinant GBA for ligand-binding studies. Taken together, our results provide a standard method for nonclinical GBA production, which should help to reduce the reliance on ERT preparations in future *in vitro* studies.

## 2. Materials and methods   

### 2.1. Generation of the recombinant transfer plasmid   

**Figure 2:**
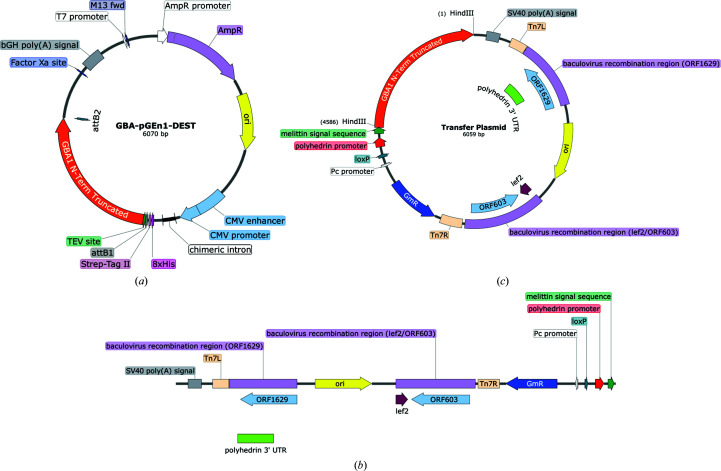
(*a*) The GBA-pGEn1-DEST plasmid containing the N-terminally truncated GBA gene. (*b*) The linearized plasmid backbone used for SLIC. (*c*) Sequenced GBA transfer plasmid generated by SLIC of the GBA (N-terminally truncated) insert and linearized plasmid backbone.

The N-terminally truncated GBA gene was subcloned from the pGEn1-GBA plasmid (DNASU Clone ID HsCD00413213; Fig. 2 ▸ *a*; gene obtained from the Glycoenzyme repository; http://glycoenzymes.ccrc.uga.edu/; Moremen *et al.*, 2018 ▸) using a Phusion (New England Biolabs) polymerase chain reaction (PCR) with the forward primer 5′-TACATTAGCTACATTTATGCGGCCCGCCCCTGCATCCCTAAAAGC-3′ and the reverse primer 5′-CTAGTACTTCTCGACAAGCTTCTACTGGCGACGCCACAGGTAG-3′.

The linearized pOMNI plasmid backbone, containing the honeybee melittin signal sequence immediately following the translation start codon (Fig. 2 ▸ *b*), was obtained by restriction digestion of an existing pOMNI plasmid using HindIII-HF and XmaI (New England Biolabs). The original pOMNI vector, containing the Tn7 transposon sequences (Tn7L and Tn7R) for Tn7 transposition, was kindly provided to the York Structural Biology Laboratory by the Berger Laboratory, University of Bristol. All DNA fragments were analysed on an agarose gel (1%) and were purified by gel extraction using a QIAquick Gel Extraction Kit (Qiagen).

The recombinant transfer plasmid was generated by sequence- and ligation-independent cloning (SLIC) of the GBA insert and linearized pOMNI backbone in One Shot TOP10 *Escherichia coli* cells (Invitrogen) using standard protocols (Li & Elledge, 2007 ▸, 2012 ▸). Briefly, the GBA insert and linearized backbone were independently treated with T4 DNA polymerase (New England Biolabs) for 30 min, followed by the addition of d-CTP. The GBA insert (1.0 ng µl^−1^) and backbone (1.5 ng µl^−1^) were treated together with RecA (New England Biolabs) in the presence of RecA buffer and ATP for 1 h at 37°C. The GBA insert and backbone were transformed into One Shot TOP10 *E. coli* cells by heat shock. The transfer-plasmid DNA was extracted and purified from overnight cultures of successful colonies in Luria–Bertani broth (LB) using a QIAprep Spin Miniprep Kit (Qiagen). The transfer plasmid was verified by restriction-digest analysis with HindIII and Sanger sequencing (Fig. 1 ▸ *c*) using the forward primer 5′-CAGCAGCGAAGTCGCCATAAC-3′ and the reverse primer 5′-CAGCCGGATCTTCTAGGCTC-3′.

### 2.2. Generation of the recombinant bacmid   

The DH10EMBacY *E. coli* strain was generously provided by the Berger Laboratory, University of Bristol. The DH10EMBacY strain contains the EMBacY baculovirus shuttle vector (bacmid bMON14272) with a mini-attTn7 target site, a tetracycline-resistant helper plasmid (pMON7124) encoding the transposase enzyme, a yellow fluorescent protein (YFP) reporter gene, the LacZα gene and a kanamycin-resistance selection marker.

The recombinant bacmid was produced using the Tn7 transposition method in DH10EMBacY cells (Bieniossek *et al.*, 2012 ▸; Fitzgerald *et al.*, 2006 ▸; Geneva Biotech). Briefly, purified transfer-plasmid DNA was transformed into DH10EMBacY cells by electroporation. Super Optimal Broth medium supplemented with 20 m*M* glucose (SOC medium) was added and the cells were incubated for 4 h at 37°C before blue/white screening on LB agar plates containing kanamycin (50 µg ml^−1^), gentamicin (15 µg ml^−1^), tetracycline (15 µg ml^−1^), IPTG (1 m*M*) and x-Gal (1×). White colonies were re-streaked and confirmed by Phusion colony PCR using the forward primer 5′-CCCAGTCACGACGTTGTAAAACG-3′ and the reverse primer 5′-AGCGGATAACAATTTCACACAGG-3′. The recombinant bacmid was purified from LB cultures of successful colonies using a PureLink HiPure Plasmid DNA Purification Kit (Invitrogen) and was verified by Phusion PCR using the forward primer 5′-CAGCAGCGAAGTCGCCATAAC-3′ and the reverse primer 5′-CAGCCGGATCTTCTAGGCTC-3′ to amplify the GBA gene and the forward primer 5′-CCCAGTCACGACGTTGTAAAACG-3′ and the reverse primer 5′-AGCGGATAACAATTTCACACAGG-3′ to amplify across the bacmid Tn7 insertion site.

### 2.3. Production of the recombinant baculovirus   

The recombinant baculovirus was generated and amplified in Sf9 cells (clonal isolate of *S. frugiperda* Sf21 cells; IPLB-Sf21-AE) purchased from Invitrogen. Adherent Sf9 cells were grown at 28°C for two days in 60 ml Insect-XPRESS protein-free medium (Lonza Bioscience) supplemented with 2% fetal bovine serum (FBS). At log-phase growth, 2 ml of suspended Sf9 cells was seeded into each well of a six-well tissue-culture plate at a density of 0.45 × 10^6^ cells ml^−1^ and allowed to settle for 10 min in a humidified incubator at 28°C to establish an adherent culture. 180 µl of a transfection mixture containing Insect-XPRESS medium (1.05 ml), recombinant bacmid DNA (∼100 µg) and FuGENE HD (Promega) transfection agent (31.5 µl) was added dropwise to each well of the six-well tissue-culture plate. The cells were incubated in a static humidified incubator at 28°C until ∼95% baculoviral transduction was achieved (∼2–3 days), as indicated by expression of the EMBacY YFP marker gene. The supernatant was collected by centrifugation at 200*g* for 5 min and FBS (0.2 ml) was added to yield the viral P1 stock. A 50 ml culture of Sf9 cells was prepared at 1 × 10^6^ cells ml^−1^ in Insect-XPRESS medium and infected with 1 ml of viral P1 stock. The culture was incubated in a shaker incubator at 28°C and 87 rev min^−1^ until 95% transfection was achieved (∼2–3 days). The supernatant was collected by centrifugation at 200*g* for 5 min and FBS (1 ml) was added to yield the viral P2 stock.

### 2.4. Expression of GBA in High Five cells   

A suspension adapted High Five cell line (BTI-Tn-5B1-4, Invitrogen) was prepared in a 60 ml culture in Express Five Serum Free Medium supplemented with 20 m*M* l-glutamine (Thermo Fisher Life Technologies). The culture was incubated at 28°C and 87 rev min^−1^ for ∼24 h. When a critical cell density (>2 × 10^6^ cells ml^−1^) had been reached, the culture was successively passaged into 100 ml, 600 ml, 1.8 l and 3.6 l Express Five Serum Free Medium. The 3.6 l culture (∼1–2 × 10^6^ cells ml^−1^) was split into 6 × 600 ml cultures and infected with 750 µl of baculovirus P2 stock. The cultures were incubated at 28°C and 87 rev min^−1^ until YFP fluorescence was observed in 95% of the cells (∼2–3 days). The supernatant was harvested by centrifugation at 400*g* for 15 min at 4°C, followed by further clearing of debris by centrifugation at 4000*g* for 60 min at 4°C. DTT and PMSF were added to achieve final concentrations of 1 and 0.1 m*M*, respectively.

### 2.5. Protein purification   

The conditioned supernatant was concentrated using a KrosFlo Research IIi Tangential Flow Filtration System with a 30 kDa mPES hollow-fibre filter module. GBA was purified using a previously outlined procedure (Sawkar *et al.*, 2006 ▸), with the addition of a size-exclusion step. Recombinant GBA was extracted from the medium by hydrophobic interaction chromatography using a TOYOPEARL Butyl-650C column (Tosoh Bioscience). The column was pre-equilibrated with 1.5 column volumes (CV) of buffer *A* (20 m*M* sodium acetate, 150 m*M* NaCl pH 5.0) and the protein was isocratically eluted into buffer *B* [20 m*M* sodium acetate, 150 m*M* NaCl, 50%(*v*/*v*) ethylene glycol pH 5.0] over 5 CV. GBA-containing fractions were pooled, diluted threefold in de­ionized water and purified by cation-exchange chromatography using a HiTrap Heparin Sepharose FF column (GE Healthcare) pre-equilibrated in buffer *A* [20 m*M* sodium acetate, 50 m*M* NaCl, 20%(*v*/*v*) ethylene glycol pH 5.0]. The protein was eluted with a linear gradient over 20 CV into buffer *B* [20 m*M* sodium acetate, 1 *M* NaCl, 20%(*v*/*v*) ethylene glycol pH 5.0]. Fractions containing GBA were pooled, diluted 15-fold in 20% ethylene glycol and purified by weak cation exchange on a HiTrap CM Sepharose FF column (GE Healthcare) pre-equilibrated with buffer *A* (30 m*M* sodium citrate, 0.01% Tween 80 pH 5.7). The protein was eluted in a linear gradient over 20 CV into buffer *B* (55 m*M* sodium citrate, 0.01% Tween 80 pH 6.3). GBA-containing fractions were pooled, concentrated to ∼1.5 ml using a 30 kDa Vivaspin concentrator (GE Healthcare) and purified using a Superdex S200 16/600 column (GE Healthcare) in SEC buffer (10 m*M* MES, 100 m*M* NaCl, 1 m*M* TCEP pH 6.5). GBA-containing fractions were concentrated to ∼10 mg ml^−1^ using a 30 kDa Vivaspin concentrator. Typical yields were 13–16.7 mg per preparation (3.6–4.6 mg per litre of culture medium). Macromolecule-production information is summarized in Table 1 ▸.

### 2.6. Michaelis–Menten kinetics   

Michaelis–Menten kinetics were assayed using the fluorogenic substrate 4-methylumbelliferyl-β-d-glucopyranoside (4-MU-Glc). GBA was prepared at 20 n*M* in kinetics buffer [McIlvaine buffer; 150 m*M* disodium hydrogen phosphate, citric acid pH 5.2 supplemented with 0.2%(*v*/*v*) taurocholate, 0.1%(*v*/*v*) Triton X-100 and 0.1%(*v*/*v*) bovine serum albumin (BSA)]. 4-MU-Glc was prepared at 5 m*M* in kinetics buffer and diluted twofold to yield solutions at 2.5, 1.25, 0.625, 0.313, 0.156, 0.078 and 0.039 m*M*. Each substrate solution (25 µl) was added to the wells of a black 384-well polystyrene plate. GBA (25 µl, 20 n*M*) was added to each well to give a final enzyme concentration of 10 n*M* and final substrate concentrations of 2.5, 1.25, 0.625, 0.313, 0.156, 0.078, 0.039 and 0.0195 m*M*. Activity against 4-MU-Glc was monitored continuously over 5 min at 37°C by measuring the fluorescence of liberated 4-MU (λ_ex_ = 360–320 nm, λ_em_ = 450–430 nm) using a CLARIOstar Plus microplate reader (BMG Labtech). Assays were performed in quadruplicate for each substrate concentration. A linear calibration was generated by measuring the fluorescence of the 4-MU product (λ_ex_ = 360–320 nm, λ_em_ = 450–430 nm) prepared at serial dilutions of 125, 62.5, 31.25, 15.63, 7.81, 3.91, 1.95 and 0.98 µ*M* in kinetics buffer. Each 4-MU concentration was measured in quadruplicate.

All data were processed using the *Origin* graphing software. Using the 4-MU calibration, the rate of substrate hydrolysis (*V*) was determined at each substrate concentration. The rates (*V*) were plotted against substrate concentration [S] and fitted by nonlinear regression to the Michaelis–Menten equation {rate = *V*_max_[S]/(*K*_m_ + [S])} to generate values of *K*_m_, *V*_max_ and *k*_cat_ using the relationship *k*_cat_ = *V*_max_/[Enz].

### 2.7. Thermal stability   

Triplicate 25 µl reactions of 2 µ*M* GBA and 5× SYPRO Orange dye (Fisher Scientific) were prepared in McIlvaine buffer at pH 5.2 and pH 7.0. The Thermofluor assay was performed using a Stratagene Mx3005P qPCR instrument. The SYPRO Orange dye was excited at λ_ex_ = 517 nm and the resulting fluorescence was monitored at λ_em_ = 585 nm with a temperature ramp from 25 to 95°C at a rate of 2°C min^−1^. Data analysis was performed using *JTSA* (http://paulsbond.co.uk/jtsa; Schulz *et al.*, 2013 ▸). The average fluorescence was plotted against temperature and fitted to a sigmoid 5 function at each pH value. The melting temperature was estimated from the midpoint of the transition.

### 2.8. Crystallization   

#### 2.8.1. Crystallization of recombinant GBA   

Purified GBA (10 mg ml^−1^) was tested against a range of commercial crystallization screens. An initial hit was found in well H8 of the PACT *premier* HT-96 screen from Molecular Dimensions (Newman *et al.*, 2005 ▸) with conditions consisting of 0.2 *M* sodium sulfate, 20%(*w*/*v*) PEG 3350, 0.1 *M* bis-Tris propane pH 8.5. Optimization of the PEG 3350 concentration and the buffer pH was performed in a 48-well MRC sitting-drop vapour-diffusion format to yield thin, rod-like crystals at pH 7 and 7.5. Further optimization of the PEG 3350 and protein concentrations resulted in larger crystals with the same morphology. The final optimized conditions were 0.3 µl GBA (10 mg ml^−1^) plus 0.5 µl well solution [0.2 *M* sodium sulfate, 14%(*v*/*v*) PEG 3350, 0.1 *M* bis-Tris propane pH 7.0].

#### 2.8.2. Sequential seeding to avoid the presence of bis-Tris propane in the active site   

As bis-Tris propane and related compounds are glycosidase inhibitors (for a review, see Roberts & Davies, 2012 ▸) that would interfere with soaking experiments, seeding was used to obtain crystals in non-bis-Tris propane conditions. Crystals obtained in the presence of bis-Tris propane were used to generate a concentrated seed stock according to previously published protocols (Shaw Stewart *et al.*, 2011 ▸). In a 48-well MRC sitting-drop vapour-diffusion format, dilutions of the concentrated seed stock (1:100 and 1:1000) were used to screen into PACT *premier* HT-96 well H8 conditions in which the bis-Tris propane had been substituted with HEPES buffer pH 7 and 7.5. Through optimization of the PEG 3350 concentration, HEPES concentration, protein volume and seeding ratios, crystals suitable for generating new seed stocks were obtained using 0.2 µl GBA (10 mg ml^−1^) plus 0.2 µl well solution [0.2 *M* sodium sulfate, 14%(*v*/*v*) PEG 3350, 0.25 *M* HEPES pH 7.0] plus 50 nl seed solution (1:1000 dilution). These seed stocks were used to rescreen previous HEPES-containing conditions in a 48-well MRC sitting-drop format, resulting in crystals that were suitable for analysis under the conditions 0.2 µl GBA (10 mg ml^−1^) plus 0.4 µl well solution [0.2 *M* sodium sulfate, 14%(*v*/*v*) PEG 3350, 0.25 *M* HEPES pH 7] plus 0.1 µl seed solution (1:1000 dilution).

#### 2.8.3. Complex with 2-deoxy-2-fluoro-β-d-glucopyranoside   

2,4-Dinitrophenyl-2-deoxy-2-fluoro-β-d-glucopyranoside (2F-DNPGlc) was prepared using well established literature protocols. Briefly, 3,4,6-tri-*O*-acetyl glucal was fluorinated using Selectfluor in acetonitrile/H_2_O (Burkart *et al.*, 1997 ▸) and was then coupled with 1-fluoro-2,4-dinitro­phenylbenzene, separated and deprotected according to published procedures (Namchuk *et al.*, 2000 ▸). Instead of peracetylation, column purification, crystallization and anomeric deacetylation to give 3,4,6-Ac-2F-Glc, DNP coupling on the Selectfluor reaction mixture was performed to yield a mixture of 3,4,6-Ac-DNP-α/β-2F-glucoside/mannoside, which were separated using 2:9:9 ethyl acetate:dichloro­methane:hexanes as the mobile phase and recrystallization.

Crystals generated in HEPES conditions following multiple rounds of seeding were soaked with 2F-DNPGlc (synthesized as above) overnight. The crystals were cryoprotected by soaking in well solution supplemented with 25% ethylene glycol prior to flash-cooling in liquid nitrogen for data collection.

#### 2.8.4. Crystallization of unliganded GBA   

An initial hit was also identified in well A5 of the JCSG-*plus* screen from Molecular Dimensions (Newman *et al.*, 2005 ▸) with conditions consisting of 0.2 *M* magnesium formate, 20%(*w*/*v*) PEG 3350. Optimization of the magnesium formate, PEG 3350 and protein concentrations resulted in larger crystals with the same morphology. The final optimized conditions were 0.6 µl GBA (10 mg ml^−1^) plus 0.5 µl well solution [0.2 *M* magnesium formate, 19%(*v*/*v*) PEG 3350].

#### 2.8.5. Cryoprotection   

All crystals were cryoprotected with well solution supplemented with 25%(*v*/*v*) ethylene glycol prior to flash-cooling in liquid nitrogen for data collection.

#### 2.8.6. Crystallization of Cerezyme   

Prior to crystallization, Cerezyme (a generous gift from Professor Hans Aerts, Leiden) was deglycosylated with PNGase F (20 µl; New England Biolabs) for five days at room temperature. The digested material was purified by size-exclusion chromatography on a Superdex 75 16/600 column. Crystals were obtained using hanging-drop vapour diffusion, based on conditions outlined by Dvir *et al.* (2003 ▸). The drops consisted of 1 µl Cerezyme (9.1 mg ml^−1^) and 1 µl well solution consisting of 1.1 *M* ammonium sulfate, 0.19 *M* guanidine–HCl, 0.04 *M* KCl, 0.1 *M* sodium acetate pH 4.6. Crystals were transferred to a lithium sulfate cryoprotectant (0.2 *M* lithium sulfate, 0.17 *M* guanidine–HCl, 0.04 *M* KCl, 0.1 *M* sodium acetate pH 4.6) before flash-cooling in liquid nitrogen.

Crystallization information is summarized in Table 2 ▸.

### 2.9. Data collection, structure solution and refinement   

Data for the bis-Tris propane complex were collected on the I04 beamline at the Diamond Light Source (DLS) and were integrated using the *DIALS* pipeline in *xia*2 (Winter, 2010 ▸; Winter *et al.*, 2018 ▸). Data reduction was performed using *AIMLESS* (Evans, 2006 ▸; Evans & Murshudov, 2013 ▸) from the *CCP*4 suite (Winn *et al.*, 2011 ▸) and the data were processed to a resolution of 1.56 Å. Molecular replacement using a previously obtained structure of Cerezyme (Artola *et al.*, 2019 ▸) as the search model was conducted with *Phaser* (McCoy *et al.*, 2007 ▸).

Data for the 2F-Glc complex were collected on the I04 beamline at DLS and were integrated using the *DIALS* pipeline in *xia*2. Data reduction was performed in *AIMLESS* and the data were processed to a resolution of 1.41 Å. The structure was solved by molecular replacement with *MOLREP* (Vagin & Teplyakov, 2010 ▸) using the bis-Tris propane complex structure obtained in this work as the search model.

Data for the unliganded crystal were collected on the I04-1 beamline at DLS and were integrated using the *autoPROC* pipeline (Vonrhein *et al.*, 2011 ▸). Data reduction was performed in *AIMLESS* and the data were processed to a resolution of 0.98 Å. Molecular replacement using PDB entry 2nt1 (Lieberman *et al.*, 2007 ▸) as the search model was conducted using *MOLREP*.

Data for the Cerezyme crystal were collected on the I03 beamline at DLS to 1.71 Å resolution and were integrated using the *DIALS* pipeline in *xia2*. The structure was solved by molecular replacement with *MOLREP* using PDB entry 2nt0 (Lieberman *et al.*, 2007 ▸) as the search model.

Data-collection and processing statistics are summarized in Table 3 ▸.

All structures were refined using *REFMAC* (Murshudov *et al.*, 2011 ▸) followed by multiple rounds of manual model building with *Coot* (Emsley *et al.*, 2010 ▸). The 0.98 Å resolution unliganded structure was anisotropically refined with multiple TLS refinement cycles using the automatic *REFMAC* option. Idealized coordinate sets and refinement dictionaries for ligands were generated using *AceDRG* (Long *et al.*, 2017*a* ▸,*b* ▸) or *JLigand* (Lebedev *et al.*, 2012 ▸). Sugar conformations were validated using *Privateer* (Agirre *et al.*, 2015 ▸), and *MolProbity* (Chen *et al.*, 2010 ▸) was used to assess model validity before deposition in the PDB. Refinement statistics are summarized in Table 4 ▸.

## 3. Results and discussion   

### 3.1. Recombinant protein production and purification   

In an attempt to circumvent the need for ERT sources of GBA for structural and biochemical studies, we sought to establish an in-house BEVS expression system for GBA. We generated a construct in which an N-terminally truncated GBA gene, lacking its native signal sequence, was used in conjunction with a honeybee melittin secretion signal (Soejima *et al.*, 2013 ▸; Tessier *et al.*, 1991 ▸) to promote the secretion of recombinant GBA into the medium. High Five cells were used for the production of recombinant GBA because they have been reported to be more efficient than Sf9 cells in secreting certain proteins (however, this effect will be protein-dependent; Wilde *et al.*, 2014 ▸).

**Figure 3:**
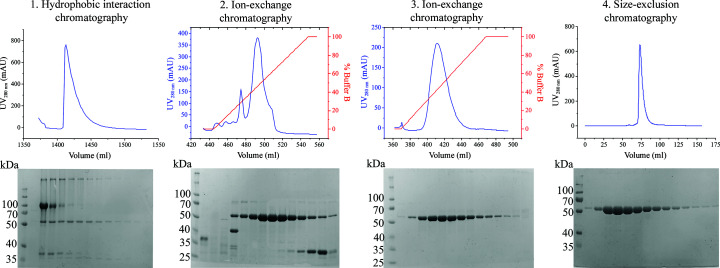
Purification of recombinant GBA from cell-culture medium with purification chromatograms and SDS–PAGE analyses for each purification step. GBA (∼55 kDa) was extracted from the medium by hydrophobic interaction chromatography followed by two rounds of cation-exchange chromatography with the addition of Tween 80 detergent. Purification was completed by a size-exclusion step to remove Tween 80.

A recombinant bacmid encoding the N-terminally truncated GBA gene was produced using the established Tn7 transposition method in DH10EMBacY cells (Bieniossek *et al.*, 2012 ▸; Fitzgerald *et al.*, 2006 ▸; Geneva Biotech; Table 1 ▸). Sf9 cells were transfected with the recombinant bacmid to generate recombinant baculovirus encoding the human GBA gene. Functional GBA was subsequently produced in High Five cells by infection with the recombinant baculovirus, resulting in secretion of the recombinant product into the cell medium. GBA was purified from the cell medium in the presence of Tween 80 detergent according to a previously outlined procedure (Sawkar *et al.*, 2006 ▸), followed by a size-exclusion step to remove the detergent and yield pure protein suitable for X-ray crystallography (Fig. 3 ▸).

Following purification, a typical yield of 3.6–4.6 mg l^−1^ was achieved, generating 13.0–16.7 mg of protein per expression. This production protocol generates sufficient purified protein for both biochemical and structural studies. Unfortunately, the study from which the purification procedure was taken failed to report a yield for comparison (Sawkar *et al.*, 2006 ▸), and only estimated yields have been provided in the very few studies in which GBA has been purified (Sinclair *et al.*, 2006 ▸). Thus, we are unable to comment on the expression yield of our GBA expression system relative to previous studies.

### 3.2. Biochemical characterization   

**Figure 4:**
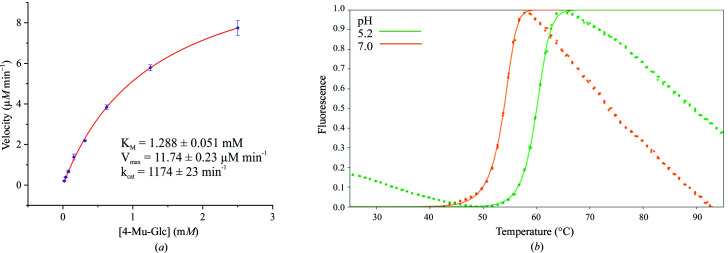
(*a*) Michaelis–Menten kinetic assay of GBA using the fluorogenic substrate 4-MU-Glc. Data are plotted as the average ± standard deviation of four replicates. (*b*) Heat-induced melting profile of GBA at pH 5.2 and pH 7.0 recorded by thermal shift assay using SYPRO Orange dye.

The biophysical properties of our GBA were investigated to evaluate whether this recombinant product could be a viable alternative to ERT formulations for nonclinical academic use. The kinetics of our GBA were assayed using the fluorogenic substrate 4-methylumbelliferyl-β-d-glucopyranoside (4-MU-Glc) and the initial reaction rates were fitted to the Michaelis–Menten equation (Fig. 4 ▸ *a*). Our recombinant enzyme exhibited comparable *K*_m_, *V*_max_ and *k*_cat_ values to those reported for Cerezyme (Tekoah *et al.*, 2013 ▸; Table 5 ▸), suggesting that our GBA produced in insect cells exhibits similar kinetic properties to Cerezyme produced in CHO cells. Furthermore, the *k*_cat_ of this recombinant enzyme compares favourably with that of GBA produced in insect cells by Sawkar *et al.* (2006 ▸) (Table 5 ▸), although no *K*_m_ or *V*_max_ values were reported in that study.

The pH-dependent stability of the recombinant protein was evaluated through a thermal shift assay at pH 5.2 and pH 7.0 (Fig. 4 ▸ *b*). Our GBA exhibited optimum stability at pH 5.2 (*T*_m_ = 60°C), as expected for an enzyme which operates in the acidic environment of the lysosome. This *T*_m_ compares favourably with that of Cerezyme (Ben Bdira *et al.*, 2017 ▸) and is 10°C higher than that of GBA produced in insect cells by Sawkar *et al.* (2006 ▸) (Table 6 ▸). The recombinant GBA demonstrated pH-dependent thermal stability, exhibiting a 6.2°C decrease in *T*_m_ at pH 7.0 compared with pH 5.2. This is consistent with the 4°C decrease that has been reported for Cerezyme (Ben Bdira *et al.*, 2017 ▸; Table 6 ▸). A decrease in thermal stability at neutral pH has been supported by proteolysis studies, in which GBA was found to be resistant to tryptic digestion at pH 5.2 but not at pH 7.4 (Ben Bdira *et al.*, 2017 ▸). This behaviour is thought to arise from changes in the native fold of the enzyme at neutral pH. Therefore, our recombinant protein exhibits the pH-dependent thermal stability profile expected for GBA. In contrast, pH-dependent thermal stability was not reported for the GBA formulation produced by Sawkar *et al.* (2006 ▸).

### 3.3. Crystallization and structure solution   

#### 3.3.1. Structure of GBA in complex with bis-Tris propane   

**Figure 5:**
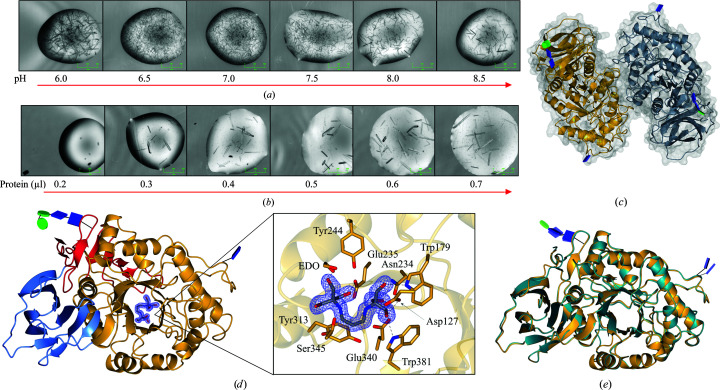
(*a*) Optimization of the crystallization pH using bis-Tris propane buffer. (*b*) Optimization of the protein concentration. (*c*) Crystal structure of the GBA dimer obtained at 1.56 Å resolution (PDB entry 6tjk). N-Glycans are depicted in glycoblock format (McNicholas & Agirre, 2017 ▸). (*d*) GBA monomer comprising of three domains: domain I (residues 1–27 and 383–414) is shown in blue, domain II (residues 30–75 and 431–497) in red and domain III (residues 76–381 and 416–430) in gold. The active site contains bound bis-Tris propane, which forms hydrogen bonds to Trp179, Asn234, Glu235, Glu340, Trp381 and an ethylene glycol (EDO) cryoprotectant molecule. Electron density is contoured to 1σ (0.34 e Å^−3^). (*e*) Overlay of recombinant GBA (gold) obtained at pH 7.0 and Cerezyme (teal) obtained at pH 4.6 (PDB entry 6tjj).

Crystals of recombinant GBA were initially obtained in well H8 [0.2 *M* sodium sulfate, 20%(*w*/*v*) PEG 3350, 0.1 *M* bis-Tris propane] of the PACT screen (Newman *et al.*, 2005 ▸) at pH 8.5. Further optimization of the buffer pH, the precipitant concentration and the protein concentration generated crystals at pH 7.0 (Figs. 5 ▸ *a* and 5 ▸ *b*), at which GBA is more active. Using 0.2 *M* sodium sulfate, 14%(*v*/*v*) PEG 3500 and 0.1 *M* bis-Tris propane (BTP) pH 7.0, GBA crystallized in space group *P*2_1_, with two molecules in the asymmetric unit, and the crystals diffracted to give a 1.56 Å resolution data set (PDB entry 6tjk; Fig. 5 ▸ *c*). Unlike in some earlier studies on GBA, we did not deglycosylate the GBA prior to crystal screening; consequently, the resulting structure exhibited visible N-glycosylation at Asn19, Asn59 and Asn146 in chain *A* ,and at Asn19 and Asn146 in chain *B*. In contrast, only glycosylation at the Asn19 site had been modelled in a number of previous studies (Dvir *et al.*, 2003 ▸; Liou *et al.*, 2006 ▸; Lieberman *et al.*, 2009 ▸). Occupancy of the Asn19 N-glycosylation site is known to be vital for GBA activity (Berg-Fussman *et al.*, 1993 ▸; Grace & Grabowski, 1990 ▸), and in this structure the Asn19 site is occupied by a chitobiose core with a β-1,4-mannose unit in chain *B* and an additional α-1,3-mannose unit in chain *A*. Only single N-linked GlcNAc residues could be modelled at the other N-glycosylation sites.

A crystal structure of Cerezyme in space group *C*222_1_ was obtained at 1.59 Å resolution (PDB entry 6tjj) for comparison with the structure of our GBA in complex with BTP. The tertiary structure of our recombinant enzyme is similar to that of Cerezyme, exhibiting the same three domains as observed in previous studies. Domain I spans residues 1–27 and 383–414, forming an antiparallel β-sheet, domain II consists of residues 30–75 and 431–497, which form an immunoglobulin-like fold, and domain III comprises a (β/α)_8_ TIM barrel formed by residues 76–381 and 416–430 (Fig. 5 ▸ *d*). There is one amino-acid change at residue 495, which is an arginine in our recombinant GBA sequence but is a histidine in Cerezyme. This is a known mutation in Cerezyme, which deviates from human placental GBA (Wei *et al.*, 2011 ▸). Overall, the tertiary structure of the recombinant GBA produced in this work compares well with the unliganded structure of Cerezyme (Fig. 5 ▸ *e*), with C^α^ root-mean-square deviations (r.m.s.d.s) of 0.57 Å (*Q*-score of 0.95) and 0.50 Å (*Q*-score of 0.96) for overlay of the *A* chains and *B* chains, respectively. However, some deviations in the protein backbone were observed in the flexible loop regions of residues 27–30, 60–64, 319–313 and 395–398. In previously published crystal structures of GBA, the loops formed by residues 311–319 and 394–399 are present in multiple conformations, suggesting dynamic flexibility of these regions. Nevertheless, this structure is also comparable with the deposited structure of Cerezyme obtained at pH 7.5 (PDB entry 2nt1; Lieberman *et al.*, 2007 ▸), with a C^α^ r.m.s.d. of ≤0.61 Å (*Q*-score of ≥0.94) for all chains.

**Figure 6:**
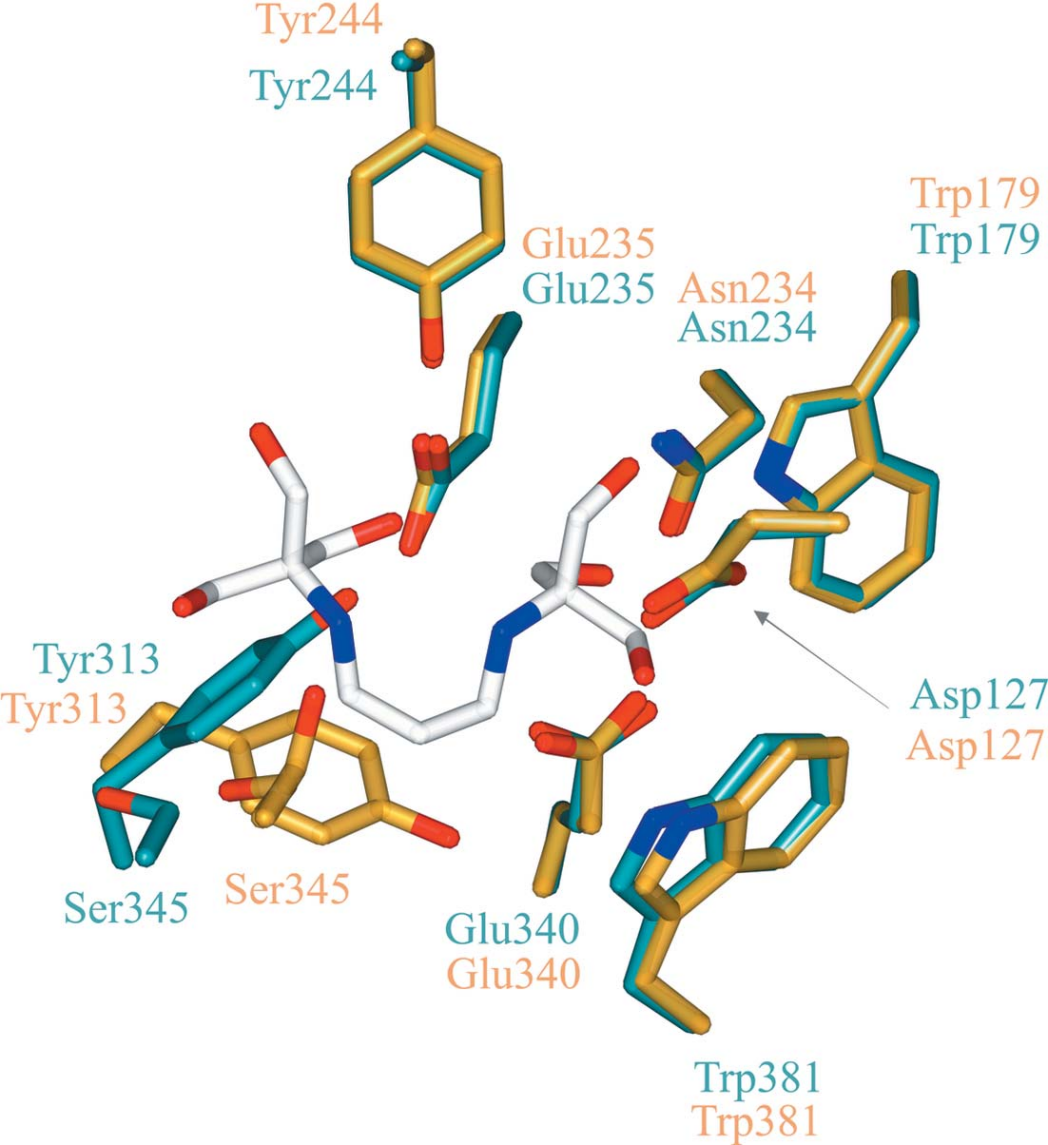
(*a*) Active-site overlay of Cerezyme (teal) and recombinant GBA (gold) with bis-Tris propane occupying the active site (white).

Unfortunately, a true ligand-free structure was not obtained owing to the binding of BTP from the crystallization conditions in the active site (Fig. 5 ▸ *d*). The binding of BTP to glycosidases has been observed previously (Thompson *et al.*, 2012 ▸; Roberts & Davies, 2012 ▸; Brunzelle *et al.*, 2008 ▸) and results from a superficial similarity between the hydroxylated and positively charged BTP molecule and the oxocarbenium-ion transition state of glycoside hydrolysis, which is strongly stabilized by glycosidase enzymes. Although the active site is occupied by BTP, when aligned with the active site of Cerezyme (Fig. 6 ▸) it is clear that most active-site residues, including the catalytic Glu235 (acid/base) and Glu340 (nucleophile), adopt almost identical conformations. However, Tyr313 is displaced downwards in the BTP complex, presumably to avoid clashing with the hydroxyl groups of the BTP molecule.

Attempts to use these crystals for ligand-binding studies by soaking with other ligands to displace BTP were unsuccessful. The inability to displace BTP from the active site can be rationalized by the high concentration of BTP used in the crystallization conditions (100 m*M*) and its comparatively potent IC_50_ (IC_50_ = 4.31 ± 0.42 m*M*) against 4-MU-Glc (Appendix *A* [App appa]). Consequently, crystals obtained under BTP-containing conditions were used for microseeding into conditions in which BTP was substituted with HEPES buffer pH 7.0. Following multiple rounds of seeding and optimization, crystals suitable for structural studies were generated using 0.2 *M* sodium sulfate, 14%(*v*/*v*) PEG 3350, 0.25 *M* HEPES pH 7.0 and 0.1 µl seed solution (1:1000 dilution).

#### 3.3.2. Trapped covalent intermediate structure   

To demonstrate the potential of these GBA crystals for use in structural studies, optimized BTP-free crystals were soaked with 2,4-dinitrophenyl-2-deoxy-2-fluoro-β-d-glucopyranoside (2F-DNPGlc) to generate a novel GBA complex structure. 2F-DNPGlc is a well characterized β-glucosidase inhibitor in which substitution of the C2 hydroxyl group with an electronegative F atom destabilizes the oxocarbenium-ion transition states for both enzyme active-site glycosylation and deglycosyl­ation (Street *et al.*, 1992 ▸; Withers *et al.*, 1988 ▸; Fig. 1 ▸). However, the addition of a reactive DNP leaving group to the aglycone increases the rate of glycosylation, allowing a trapped enzyme–inhibitor complex to accumulate after reaction with the enzyme (Withers *et al.*, 1988 ▸). Activated 2-deoxy-2-fluoroglycosides have been used in combination with X-ray crystallography to gain mechanistic insights into retaining glycosidases, with 2-deoxy-2-fluoro-β-d-glucopyranosyl fluoride notably having been used to correct the identity of the catalytic nucleophile of GBA (Miao *et al.*, 1994 ▸), but no co-crystal complex of GBA with this inhibitor has been previously reported.

**Figure 7:**
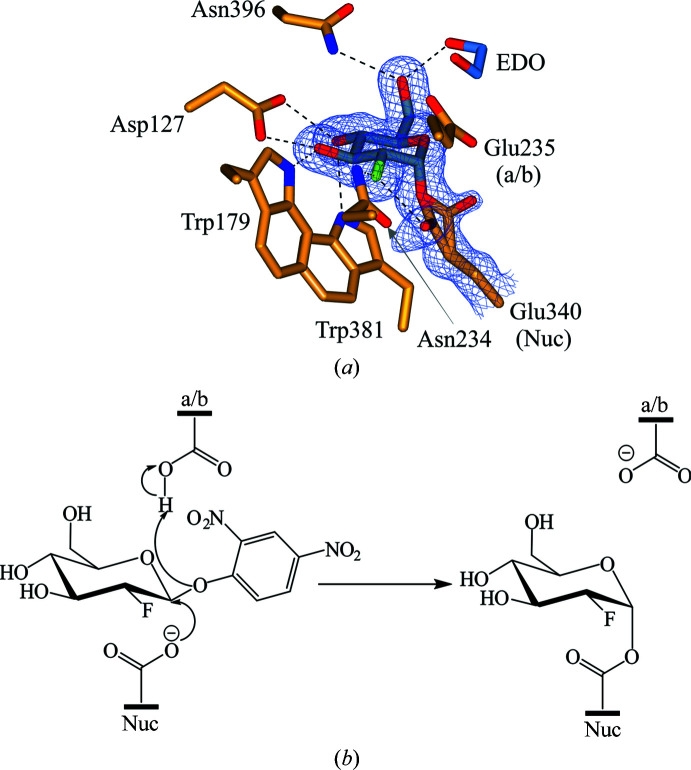
(*a*) Active-site structure of the 2-deoxy-2-fluoro-β-d-glucopyranoside-GBA covalent intermediate (PDB entry 6tjq). The 2F-Glc moiety is covalently bound to the catalytic nucleophile (Glu340), which occupies two conformations. a/b, catalytic acid/base; Nuc, catalytic nucleophile; EDO, ethylene glycol. Electron density is contoured to 1.1σ (0.40 e Å^−3^). (*c*) Mechanism of the hydrolysis of 2F-DNPGlc by GBA to generate the covalent glycosyl-enzyme intermediate

A structure of the 2-deoxy-2-fluoroglucopyranosyl-GBA intermediate was obtained at 1.41 Å resolution (PDB entry 6tjq), showing unambiguous electron density for covalent binding of the 2-deoxy-2-fluoroglucose moiety to the catalytic nucleophile, with a covalent bond length of 1.42 Å (Fig. 7 ▸ *a*). The glucose-configured ring adopts a ^4^ *C*_1_ chair conformation, consistent with the conformation of the covalent glycosyl-enzyme intermediate in the GBA conformational itinerary (Fig. 1 ▸). The bound 2F-glycone moiety also forms hydrogen bonds to Trp179, Asp127, Trp179, Asn234, Glu340, Trp381, Asn396 and an ethylene glycol cryoprotectant molecule. Interestingly, two conformations of the catalytic nucleophile can be observed (Fig. 7 ▸ *a*). We postulate that electrostatic repulsion between the carboxylate of the catalytic nucleophile and the C2-linked F atom of the 2F-Glc inactivator enforces a 28° rotation about C^γ^ of the nucleophilic residue, resulting in movement of the O1 atom of the carboxylate residue away from the C2-linked F atom. Aside from providing a novel structure in complex with a mechanistically relevant glucosidase inhibitor, this complex demonstrates the ability of our GBA to be used as an alternative to ERT preparations in the structure-based development of new inhibitory compounds for GBA.

#### 3.3.3. Atomic resolution ligand-free structure   

**Figure 8:**
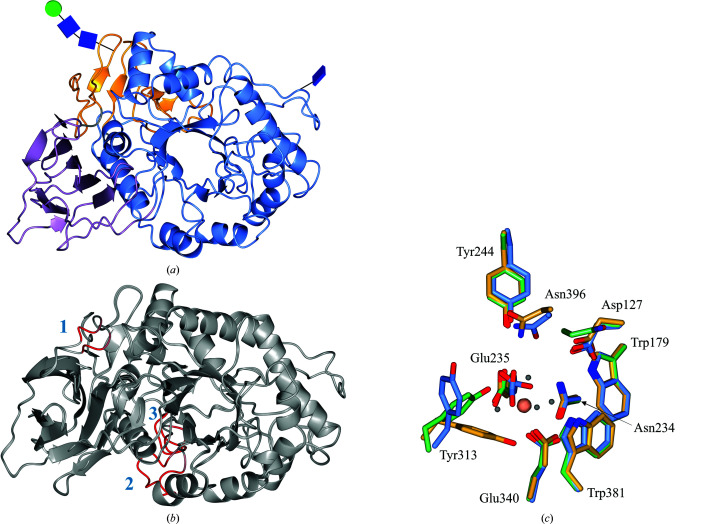
(*a*) Crystal structure of the GBA monomer obtained at 0.98 Å resolution (PDB entry 6tn1). Domain I (residues 1–27 and 383–414) is shown in lilac, domain II (residues 30–75 and 431–497) in orange and domain III (residues 76–381 and 416–430) in blue. N-Glycans are depicted in glycoblock format (McNicholas & Agirre, 2017 ▸). (*b*) Overlay of the unliganded GBA structure with the BTP-complexed structure (PDB entry 6tjk). Red indicates areas of high r.m.s.d. between the protein backbones. Loop 1 contains residues 27–31, loop 2 comprises residues 314–319 and loop 3 contains residues 344–350. (*c*) Active site of the unliganded GBA crystal structure (blue) overlaid with active-site residues of the BTP-complex structure (gold; PDB entry 6tjk) and Cerezyme (green; PDB entry 6tjj). A magnesium ion (peach) coordinated by four waters (grey), Glu340 (Nuc) and Glu235 (a/b) occupies the active site.

Given the tight binding of BTP in our originally identified GBA crystal form, we also sought to identify non-BTP-containing crystallization conditions in parallel with our efforts to remove BTP by microseeding. During initial screening, crystals were also found under condition A5 [0.2 *M* magnesium formate, 20%(*w*/*v*) PEG 3350] of the JCSG-*plus* screen (Newman *et al.*, 2005 ▸). Optimized crystals suitable for structural analysis were obtained using 0.2 *M* magnesium formate, 19%(*v*/*v*) PEG 3350. Subsequently, a 0.98 Å resolution unliganded structure of GBA was obtained (PDB entry 6tn1). Not only is this the highest resolution structure of GBA deposited to date, it also exists in a previously unreported crystal form. Previously, GBA has been crystallized in space groups *C*222_1_ and *P*2_1_; however, this unliganded structure crystallized in space group *P*1. The new structure contains one molecule in the asymmetric unit, which comprises three noncontiguous domains, with N-glycosylation at Asn19 and Asn146 (Fig. 8 ▸ *a*). Overall, the three-domain tertiary structure is highly similar to that of the BTP complex and Cerezyme, with C^α^ r.m.s.d.s of 0.49 Å (*Q* score of 0.94) and 0.60 Å (*Q* score of 0.94), respectively. However, some deviations in the protein backbone were observed in the flexible loop regions consisting of residues 26–31, 314–319 and 344–350 (Fig. 8 ▸ *b*). Despite the sub-Ångström resolution, residues 26–31 and 314–319 were challenging to model, reflecting the flexibility and disorder of these loops, which has also been observed in previous GBA structures. Importantly, the active site of this unliganded structure compares well with the active sites of Cerezyme and the BTP complex. The majority of active-site residues occupy essentially identical conformations, with the exception of Tyr313, which restores its ‘upwards’ conformation in the absence of BTP (Fig. 8 ▸ *c*). In fact, Tyr313 appears to be particularly mobile, occupying a different conformation in each GBA structure.

**Figure 9:**
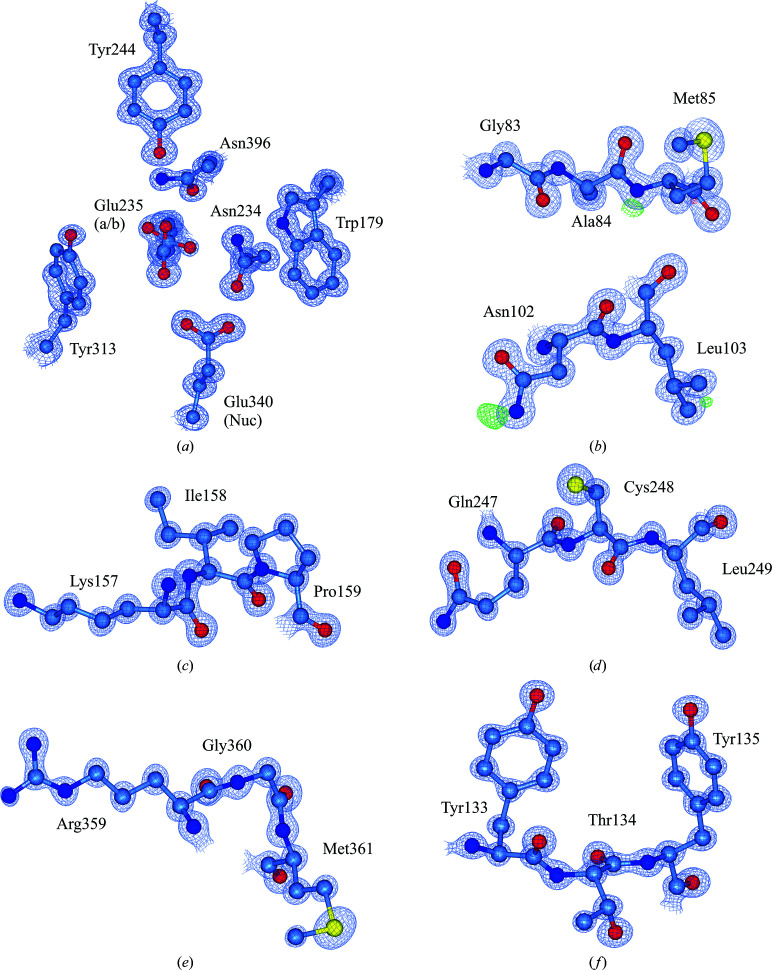
(*a*) Electron density for active-site residues, including the catalytic nucleophile (Nuc) Glu340 and catalytic acid/base (a/b) Glu235, contoured to 2σ (1.0 e Å^−3^). (*b*) Selection of modelled residues with difference electron density [green; contoured to 3σ (0.37 e Å^−3^)] highlighting proton positions. (*c*)–(*f*) Modelled residues from domain I of GBA (PDB entry 6tn1) demonstrating atomic resolution (electron density contoured to 3.5σ (1.75 e Å^−3^).

The sub-Ångström resolution of this unliganded structure permits the first ever atomic resolution analysis of GBA, uncovering finer details in its structure. For example, two conformations of the catalytic acid/base residue (Glu235) can be observed (Fig. 9 ▸ *a*). In fact, many alternative side-chain conformations could be modelled throughout the structure, providing more detail on side-chain mobility and interactions. We also observed proton positions for some residues in the difference electron density, as well as electron delocalization over carbonyls and double bonds (Figs. 9 ▸ *b*–9 ▸ *f*). We anticipate that this new crystal form will be utilized in structural studies to provide atomic resolution analysis of ligand binding and interactions with GBA.

## 4. Conclusions   

This work describes a detailed approach to the production of active human GBA in an AcNPV-derived baculoviral expression system, providing an alternative source to ERT formulations. Recombinant GBA was produced and secreted from baculovirus-transduced insect cells by the action of the honeybee melittin signal sequence, and was purified with a typical yield of 3.6–4.6 mg l^−1^. The recombinant protein was shown to be active against the artificial substrate 4-methyl­umbelliferyl-β-d-glucopyranoside (*K*_m_ = 1.3 m*M*, *k*_cat_ = 1174 min^−1^) and exhibited optimum thermal stability at pH 5.2 (*T*_m_ = 60°C), consistent with the biophysical properties of the commercial ERT Cerezyme. Moreover, our recombinant GBA crystallizes readily and is amenable to structural ligand binding, as demonstrated by a novel structure in complex with the glucosidase inhibitor 2,4-dinitrophenyl-2-deoxy-2-fluoro-β-d-glucopyranoside. We also identified a novel crystal form of GBA which diffracts to 0.98 Å resolution. This is the highest resolution structure of GBA deposited to date, permitting exquisite atomic resolution analysis. Multiple alternative residue conformations were observed throughout the structure, including two conformations of the catalytic acid/base residue. We envision that the BEVS GBA production system described in this work will alleviate the over-reliance on ERT formulations, aid in future biochemical studies of GBA and support structure-guided development of novel GBA ligands.

## Supplementary Material

PDB reference: β-glucocerebrosidase, 6tn1

PDB reference: complex with bis-Tris propane, 6tjk

PDB reference: complex with 2,4-dinitrophenyl-2-deoxy-2-fluoro-β-d-glucopyranoside, 6tjq

PDB reference: Cerezyme, 6tjj

## Figures and Tables

**Figure 10:**
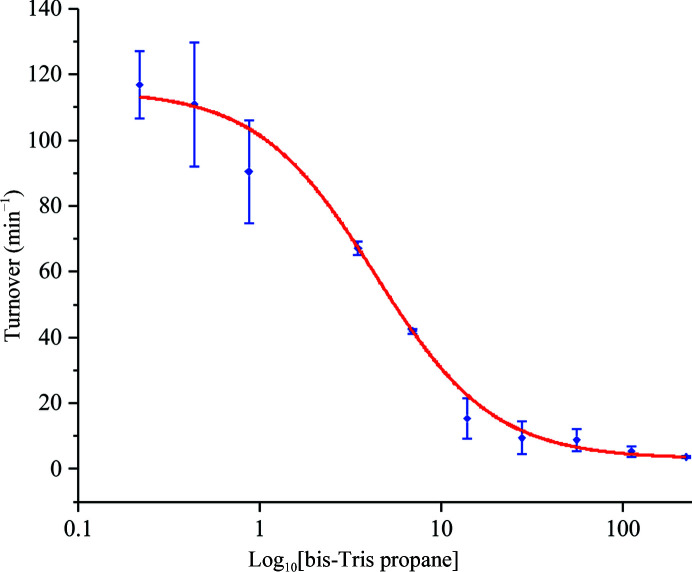
Rate of hydrolysis of 4-MU-Glc at pH 7.0 versus bis-Tris propane concentration fitted to a four-parameter logistic function to estimate the IC_50_ of bis-Tris propane.

**Table 1:** Macromolecule-production information The 5′ end of the forward primer was designed to be complementary to the melittin signal sequence of the linearized p-OMNI backbone and the 5′ end of the reverse primer was designed to be complementary to the multi-insertion site of the linearized p-OMNI backbone (denoted in bold).

Source organism	*Homo sapiens*
DNA source	GBA-pGEn (DNASU: HsCD00413213; Moremen *et al.*, 2018 ▸)
Forward primer	**TACATTAGCTACATTTATGCG**GCCCGCCCCTGCATCCCTAAAAGC
Reverse primer	**CTAGTACTTCTCGACAAGCTT**CTACTGGCGACGCCACAGGTAG
Cloning vector	pOMNI-derived vector (Sari *et al.*, 2016 ▸)
Expression vector	DH10EMBacY AcNPV-derived vector (Fitzgerald *et al.*, 2006 ▸)
Expression host	*Trichoplusia ni* (BTI-Tn-5B1-4, High Five cells)
Complete amino-acid sequence of the construct produced	ARPCIPKSFGYSSVVCVCNATYCDSFDPPTFPALGTFSRYESTRSGRRMELSMGPIQANHTGTGLLLTLQPEQKFQKVKGFGGAMTDAAALNILALSPPAQNLLLKSYFSEEGIGYNIIRVPMASCDFSIRTYTYADTPDFQLHNFSLPEEDTKLKIPLIHRALQLAQRPVSLLASPWTSPTWLKTNGAVNGKGSLKGQPGDIYHQTWARYFVKFLDAYAEHKLQFWAVTAENEPSAGLLSGYPFQCLGFTPEHQRDFIARDLGPTLANSTHHNVRLLMLDDQRLLLPHWAKVVLTDPEAAKYVHGIAVHWYLDFLAPAKATLGETHRLFPNTMLFASEACGSKFWEQSVRLGSWDRGMQYSHSIITNLLYHVVGWTDWNLALNPEGGPNWVRNFVDSPIIVDITKDTFYKQPMFYHLGHFSKFIPEGSQRVGLVASQKNDLDAVALMHPDGSAVVVVLNRSSKDVPLTIKDPAVGFLETISPGYSIHTYLWRRQ

**Table 2:** Crystallization

	PDB entry 6tjk	PDB entry 6tjj	PDB entry 6tjq	PDB entry 6tn1
Method	Sitting-drop vapour diffusion	Hanging-drop vapour diffusion	Sitting-drop vapour diffusion	Sitting-drop vapour diffusion
Plate type	MRC Maxi 48-well	24-well XRL	MRC Maxi 48-well	MRC Maxi 48-well
Temperature (K)	293	293	293	293
Protein concentration (mg ml^−1^)	10	10	10	10
Buffer composition of protein solution	10 m*M* MES, 100 m*M* NaCl, 1 m*M* TCEP pH 6.5	20 m*M* MES, 100 m*M* NaCl pH 6.5	10 m*M* MES, 150 m*M* NaCl, 1 m*M* TCEP pH 6.5	10 m*M* MES, 100 m*M* NaCl, 1 m*M* TCEP pH 6.5
Composition of reservoir solution	0.2 *M* Na_2_SO_4_, 14%(*v*/*v*) PEG 3350, 0.1 *M* bis-Tris propane pH 7.0	1.1 *M* (NH_4_)_2_SO_4_, 0.19 *M* guanidine–HCl, 0.04 *M* KCl, 0.1 *M* sodium acetate pH 4.6	0.2 *M* Na_2_SO_4_, 14%(*v*/*v*) PEG 3350, 0.25 *M* HEPES pH 7.0	0.2 *M* magnesium formate, 19%(*v*/*v*) PEG 3350
Volume and ratio of drop (protein:well) (µl)	0.8 (0.3:0.5)	2 (1:1)	0.7 (0.2:0.4, + 0.1 seed)	1.1 (0.6:0.5)
Volume of reservoir (µl)	100	500	100	100

**Table 3:** Data collection and processing Values in parentheses are for the outer resolution shell.

	PDB entry 6tjk	PDB entry 6tjj	PDB entry 6tjq	PDB entry 6tn1
Diffraction source	Beamline I04, DLS	Beamline I03, DLS	Beamline I04, DLS	Beamline I04-1, DLS
Wavelength (Å)	0.9795	0.9763	0.9795	0.9159
Temperature (K)	100	100	100	100
Detector	EIGER2 XE 16M	EIGER2 XE 16M	EIGER2 XE 16M	PILATUS 6M-F
Rotation range per image (°)	0.1	0.1	0.1	0.1
Total rotation range (°)	360	360	360	360
Exposure time per image (s)	0.020	0.020	0.010	0.040
Space group	*P*2_1_	*C*222_1_	*P*2_1_	*P*1
*a*, *b*, *c* (Å)	52.7, 156.2, 68.3	110.1, 285.9, 91.9	53.1, 76.4, 68.2	44.5, 46.2, 64.2
α, β, γ (°)	90, 102, 90	90, 90, 90	90, 102, 90	86, 75, 83
Resolution range (Å)	66.75–1.56 (1.59–1.56)	77.44–1.59 (1.62–1.59)	52.01–1.41 (1.43–1.41)	31.70–0.98 (1.00–0.98)
Total No. of reflections	637325 (31169)	1531796 (74996)	689440 (33887)	394040 (1815)
No. of unique reflections	147746 (7162)	193710 (9517)	102363 (5031)	208682 (1518)
Completeness (%)	96.8 (94.7)	100 (100)	100 (99.7)	74.2 (10.9)†
Multiplicity	4.3 (4.4)	7.9 (7.9)	6.7 (6.7)	1.9 (1.2)
〈*I*/σ(*I*)〉	7.3 (0.7)	7.2 (0.5)	7.2 (0.4)	8.7 (1.0)
*R*_p.i.m._	0.057 (0.90)	0.055 (2.34)	0.056 (1.66)	0.037 (0.49)
CC_1/2_	0.99 (0.34)‡	0.99 (0.68)	0.99 (0.36)‡	0.99 (0.69)
Overall *B* factor from Wilson plot (Å^2^)	18	24	18	7

† The low completeness for the outer resolution shell (also reflected in the overall statistics) for PDB entry 6tn1 reflected integration into the corners of a square detector.

‡ Data with a low outer bin *I*/σ(*I*) (with a CC_1/2_ of approximately 0.35) were used as they were reflected by improved maps with appropriate model-refinement statistics at these resolutions.

**Table 4:** Structure solution and refinement Values in parentheses are for the outer resolution shell.

	PDB entry 6tjk	PDB entry 6tjj	PDB entry 6tjq	PDB entry 6tn1
Resolution range (Å)	66.75–1.56 (1.59–1.56)	77.44–1.59 (1.62–1.59)	52.01–1.41 (1.43–1.41)	31.70–0.98 (1.00–0.98)
Completeness (%)	96.8 (94.7)	100 (100)	100 (99.7)	74.2 (10.9)
Final *R*_cryst_	0.17	0.22	0.18	0.11
Final *R*_free_	0.20	0.25	0.21	0.13
Cruickshank DPI (Å)	0.08	0.09	0.06	0.02
No. of non-H atoms				
Protein	7987	8008	4065	4486
Ion	—	2	—	8
Ligand	418	242	173	167
Water	907	721	484	709
Total	9312	8973	4722	5370
R.m.s. deviations				
Bonds (Å)	0.014	0.014	0.013	0.009
Angles (°)	1.80	1.84	1.71	1.61
Average *B* factors (Å^2^)				
Protein	23	34	21	8
Ion	—	42	—	16
Ligand	39	67	42	15
Water	35	42	35	29
Ramachandran plot				
Most favoured (%)	95	95	95	95
Allowed (%)	4	4	4	4

**Table 5:** Kinetic analysis of recombinant GBA and comparison with Cerezyme and with GBA produced in insect cells by Sawkar *et al.* (2006 ▸)

	Recombinant GBA†	Cerezyme‡	rhWT-GBA§
*K*_m_ (m*M*)	1.288 ± 0.051	1.127 ± 0.052	—
*V*_max_ (µ*M* min^−1^)	11.74 ± 0.23	2.21 ± 0.03	—
*k*_cat_ (min^−1^)	1174 ± 23	1325 ± 2	868 ± 28

† Values for recombinant GBA produced in this study. The data shown are the average ± standard deviation of four replicates.

‡ Values reported for Cerezyme (Tekoah *et al.*, 2013 ▸).

§ *k*_cat_ value for GBA produced in insect cells by Sawkar *et al.* (2006 ▸) determined by conversion of the reported specific activity.

**Table 6:** *T*_m_ values for the thermal denaturation of recombinant GBA, Cerezyme (Lieberman *et al.*, 2009 ▸) and rhWT-GBA produced in insect cells by Sawkar *et al.* (2006 ▸)

Protein	*T*_m_ (°C)	
Recombinant GBA†	60.0 ± 0.2 (pH 5.2)	53.8 ± 0.1 (pH 7.0)
rhWT-GBA‡	49.3 (pH 5.3)	49.2 (pH 7.0)
Cerezyme§	61 (pH 5.2)	57 (pH 7.4)

† *T*_m_ values of recombinant GBA produced in this work as determined by a Thermofluor assay. Data are reported as the average ± standard deviation of three replicates.

‡ *T*_m_ values of GBA produced in insect cells by Sawkar *et al.* (2006 ▸) as determined by circular dichroism (Sawkar *et al.*, 2006 ▸).

§ *T*_m_ values reported for Cerezyme as determined by circular dichroism (Ben Bdira *et al.*, 2017 ▸).

## Appendix A The IC_50_ of bis-Tris propane

The IC_50_ of bis-Tris propane was determined using the fluorogenic substrate 4-methylumbelliferyl-β-d-glucopyranoside (4-MU-Glc). GBA was prepared at 58 n*M* in kinetics buffer [McIlvaine buffer; 150 m*M* disodium hydrogen phosphate, citric acid pH 7.0 supplemented with 0.2%(*v*/*v*) taurocholate, 0.1%(*v*/*v*) Triton X-100 and 0.1%(*v*/*v*) bovine serum albumin (BSA)]. Bis-Tris propane was prepared at 500 m*M* in kinetics buffer pH 7.0 and diluted twofold in serial dilutions to 0.488 µ*M*. The solutions (20 µl) were added to the wells of a black 384-well polystyrene plate followed by the addition of 4-MU-Glc (20 µl) prepared at 4 m*M* in kinetics buffer. GBA (5 µl) was added to each well to yield a final enzyme concentration of 6.5 n*M*. The activity against 4-MU-Glc was monitored continuously over 5 min at room temperature by measuring the fluorescence of liberated 4-MU (λ_ex_ = 360–320 nm, λ_em_ = 450–430 nm) using a CLARIOstar Plus micro­plate reader (BMG Labtech). The assays were performed in quadruplicate for each bis-Tris propane concentration. A linear calibration was generated by measuring the fluorescence of the 4-MU product (λ_ex_ = 360–320 nm, λ_em_ = 450–430 nm) prepared at serial dilutions of 62.5, 31.25, 15.63, 7.81, 3.91 and 1.95 µ*M* in kinetics buffer. The data were processed using the *Origin* graphing software. Using the 4-MU calibration, the rate of substrate hydrolysis (*V*) was determined at each concentration of bis-Tris propane. The rates (*V*) were plotted against the log of the bis-Tris propane concentration and were fitted by nonlinear regression to the four-parameter logistic function to determine the IC_50_ (IC_50_ = 4.31 ± 0.42 m*M*; Fig. 10 ▸).
